# Optimizing Titanium Osseointegration through Thermally Modified Co-Doped Monetite Coatings

**DOI:** 10.1021/acsbiomaterials.5c01648

**Published:** 2026-02-16

**Authors:** Gerson Santos de Almeida, Maria Gabriela Jacheto Carra, Matheus Luquirini Penteado dos Santos, Julia Bucci, Luisa Camilo Suter, Diego Rafael Nespeque Corrêa, Pascale Chevalier, Margarida Juri Saeki, Diego Mantovani, Willian Fernando Zambuzzi

**Affiliations:** † Lab. of Bioassays and Cell Dynamics, Department of Chemistry and Biochemistry, Institute of Biosciences, 164767 UNESP—Universidade Estadual Paulista, Botucatu, São Paulo 18618-970, Brazil; ‡ Lab. of Materials and Electrochemistry, Department of Chemical and Biochemistry, Institute of Biosciences, UNESP—Universidade Estadual Paulista, Botucatu, São Paulo 18618-970, Brazil; § Laboratory of Anelasticity and Biomaterials, Department of Physics and Meteorology, School of Sciences, São Paulo State University—UNESP, Bauru, São Paulo 17033-360, Brazil; ∥ Laboratory for Biomaterials and Bioengineering, Department of Min-Met-Materials Engineering and Regenerative Medicine, & Regenerative Medicine, CHU de Quebec Research Center, 177453 Laval University, Quebec City G1L 3L5, Canada; ⊥ INCT-BIOCRON, UNESP: Universidade Estadual Paulista, Botucatu, Sao Paulo 18603-100, Brazil

**Keywords:** monetite, cobalt-doped monetite, titanium implants, hydrothermal coatings, osseointegration, angiogenesis

## Abstract

Although Ti implants have been used clinically for decades, their osseointegration is still a major concern in aged, diseased and osteoporotic patients. Using a hydrothermal synthesis approach, monetite (CaHPO_4_) and Co-monetite coatings with controlled crystallinity and surface topography were designed and produced. Structural characterization via X-ray diffraction (XRD) confirmed the formation of phase-pure monetite (triclinic) with homogeneous cobalt distribution, while scanning electron microscopy (SEM) and profilometry revealed microstructured surfaces featuring peaks and valleys, mimicking native bone morphology. Remarkably, the coatings exhibited superhydrophilic properties for Co-monetite versus uncoated Ti. Biological assessments demonstrated excellent cytocompatibility using preosteoblasts, with MTT assays showing higher metabolic activity in Co-monetite groups compared to control. SEM analysis revealed enhanced preosteoblast adhesion and spreading on Co-monetite surfaces by day 7. Gene expression profiling uncovered significant upregulation of osteogenic markers, while zymography further demonstrated increased both MMP-2/9 activity, indicating active extracellular matrix remodeling. Altogether, these findings highlight the dual functionality of Co-monetite coatings toward (1) the physicochemical properties that promote osteoblast adhesion and early differentiation, and (2) cobalt doping, that induces a pro-angiogenic response through HIF-1α stabilization. By addressing both osteogenesis and vascularization, two critical challenges in implant integration, this research provides a foundation for the rational design of multifunctional biomaterial coatings for orthopedic and dental applications. The results suggest that Co-monetite coatings are a promising strategy to enhance the osseointegration of bone implants, warranting further preclinical investigation.

## Graphical abstract

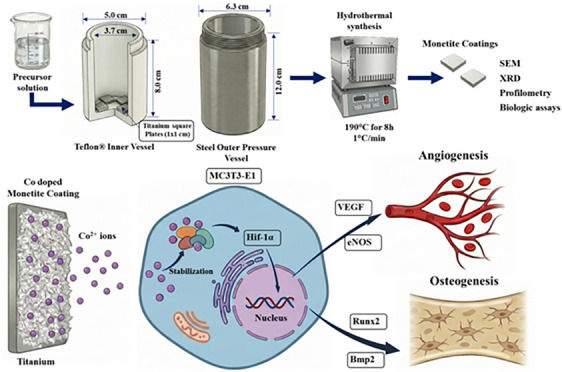

## Introduction

Titanium (Ti) has underpinned skeletal reconstruction since the 1960s, following Brånemark’s pioneering work, due to its biocompatibility, mechanical strength, and corrosion resistance.^1^ Despite its status as the gold standard, relevant clinical limitations persist—particularly delayed osseointegration and peri-implant inflammation.^2,3^ Consequently, surface modification is essential. Among available strategies, calcium phosphate (CaP) coatings—especially hydroxyapatite (HA) and monetite—are prominent owing to their chemical similarity to bone mineral and robust osteoconductivity.^4,5^ Monetite (CaHPO_4_) offers additional advantages over HA, including higher solubility and bioresorption, which may accelerate bone remodeling and implant anchorage.^6–9^ In this context, bioactive coatings have emerged as a leading approach to mitigate early failures,^10–14^ with CaPs remaining attractive candidates to enhance bone–implant integration.^4,5,15,16^ Monetite, in particular, has garnered interest for its bioactivity and inherent resorbability, supporting physiological remodeling at the interface.^6,17^

Building on this rationale, we investigate monetite and cobalt-doped monetite (Co-monetite) coatings on Ti. Cobalt is selected for its angiogenic action and capacity to mimic hypoxic cues, thereby favoring osteogenesis–angiogenesis coupling.^9,18^ Recent studies indicate that Co^2+^ can foster endothelial recruitment and collagen deposition, events critical to early bone healing.^19–21^ Nonetheless, the combined effects of Co-monetite on Ti—especially its influence on osteoblast–ECM crosstalk and early molecular programs—remain underexplored.

Although Ti implants have been clinically implanted for decades, their osseointegration is still a major concern in aged, diseased and osteoporotic patients. Here, we synthesize monetite and Co-monetite coatings via a low-temperature hydrothermal route that yields crystalline, high-purity layers while preserving the integrity of the Ti substrate. We integrate physicochemical characterization with biological assays to relate composition and surface attributes (roughness, wettability) to cellular responses, and we probe mechanistic pathways through gene-expression profiling (e.g., Runx2, VEGF, MMPs). We hypothesize that Co-monetite coatings will enhance early osseointegration by providing a dual stimulus: (1) physicochemical cues from the monetite matrix that promote osteoblast adhesion and early differentiation, and (2) cobalt doping that induces a pro-angiogenic response, thereby coupling osteogenesis and vascularization.

## Experimental Section

The methodology employed was adapted from the protocol proposed by Zhou et al.,^1^ utilizing hydrothermal treatment for the coatings. To coat with monetite, reagents were used at the following concentrations: 0.2 M CaCl_2_, 0.12 M NaH_2_PO_4_, and 0.1 M Na_2_EDTA-2H_2_O, dissolved in deionized water. The solution was adjusted to a final pH of 3.4–3.6 by carefully adding HCl or NH_4_OH. For the Co-monetite coating, the concentrations of all reagents remained the same, except for calcium and cobalt, which were present at 0.18 M and 0.02M, respectively. Commercially pure titanium (cp-Ti, ASTM F67 GR2) served as the substrate; it was abraded and then treated with a 10% hydrochloric acid solution. For the coating process, commercially pure titanium (c.p.-Ti) plates and the prepared precursor solution were placed into a custom-designed hydrothermal reactor. The system comprised an inner Teflon (PTFE) reaction vessel with dimensions of 5.0 × 8.0 cm^2^, sealed with metal disc gaskets, and housed within an outer stainless-steel pressure vessel measuring 6.3 × 12.0 cm^2^. All components of the reactor assembly were designed and manufactured by the research group and are not commercially available. Once loaded, the reactor was placed into a muffle furnace (EDG, F3000) and heated at a rate of 1 °C/min until reaching the target temperature of 190 °C. The hydrothermal treatment was maintained for 8 h to promote the formation of the coating under controlled temperature and pressure conditions. Upon completion of the reaction, the system was allowed to cool naturally to room temperature. The coated titanium plates were then carefully retrieved, rinsed thoroughly with deionized water to remove any residual reactants, and subjected to a subsequent thermal treatment at 200 °C for 2 h in air, with a heating rate of 15 °C/min. This postsynthesis heat treatment was performed to improve the crystallinity and phase stability of the deposited monetite layer, as reported in similar procedures in the literature. After coating, the samples were characterized by XRD, SEM/EDX, profilometry, contact angle, and cytotoxicity tests (MTT and CV), the morphology of the MC3T3-E1 cells was evaluated by SEM, gene expression was evaluated by RTqPCR and metalloproteinase activity by zymography.

### X-ray Diffraction (XRD)

The applied coatings underwent crystalline structure analysis utilizing a RIGAKU X-ray diffractometer (DMAX-2100), employing Cu–Kα radiation at a wavelength of 1.5418 Å. This was performed under a potential of 40 kV and a current of 20 mA, utilizing a grazing incidence angle of 2°. The diffractograms were recorded over a 2θ range from 10° to 80°, with a step size of 0.020° and a dwell time of 1 s per step. Phase identification was conducted through comparison with standard references from the Powder Diffraction File (PDF-2003) and the Inorganic Crystal Structures Database (ICSD, Version 2.2), using Crystallographica Search Match software for assistance. Structural parameters and the degree of crystallinity were refined using the Rietveld method through GSAS software and the EXPGUI graphical user interface. Instrumental broadening corrections were applied using a standard Y_2_O_3_ sample, and the profiles were adjusted using functions such as Pseudo-Voigt for peak shapes, Chebyshev for background subtraction, and March-Dollase for preferred orientation correction. The goodness-of-fit (GoF) and R’s merit parameters for successful refinement were considered the same as those from our previous paper.^2^ Calculations of the crystallite size were based on the full-width at half-maximum (fwhm) of the peaks, applying both Scherrer and Williamson-Hall equations.^3–6^

### Scanning Electron Microscopy (SEM)

Observations of the surface morphology and cellular interactions on the titanium coatings were conducted with a FEI Quanta 200 scanning electron microscope. For the coatings, a magnification of 2000× was used with an accelerating voltage of 12.5 kV. When examining cellular morphology, the magnification was increased to 5000×, under the same voltage, to capture finer details. The semiquantitative EDS analysis was performed in the same equipment for Ca, P, O, and Co chemical elements using the same acceleration voltage (10 kV).

### Contact Angle

Wettability assessments were performed using the VCA 2500 XE system to measure the static contact angle at ambient conditions. Droplets of 1 μL of ultrapure water were delicately placed on three separate areas of each specimen, with this process repeated across three individual samples to ensure reliability of the data. Due to the immediate and complete spreading of water droplets on the CaP and CoCaP coatings, the measured contact angles (∼11° and ∼10°) should be interpreted as indicative of extreme hydrophilicity rather than precise equilibrium values.

### Profilometry

The topographical attributes, specifically the roughness of both uncoated and coated titanium samples, were quantified through profilometry. Employing a Bruker Dektak XT Profilometer, we scanned a surface area of 1 mm by 1 mm, utilizing a stylus with a tip radius of 12.5 μm and applying a force of 3 mg to trace the surface contours. The average roughness (*R*_a_) and root-mean-square roughness (*R*_q_) were calculated from three independent scans per sample group.

### Osteoblasts Culture

The MC3T3-E1 preosteoblast cell line (ATCC CRL-2593) was used between passages 15 and 25. Cells were routinely tested and confirmed to be free of mycoplasma contamination by qPCR. Cells were propagated in α-MEM enriched with a blend of antibiotics (penicillin at 100 units/mL and streptomycin at 100 mg/mL) and fortified with 10% fetal bovine serum (FBS). Incubation was carried out at 37 °C, ensuring a humidified atmosphere with 95% relative humidity and a 5% CO_2_ environment. Conditioned media were prepared according to ISO 10993–12:2021, using the indirect contact method for solid samples. Cp-Ti, CaP-Ti, and CoCaP-Ti disks (10 mm diameter × 2 mm thickness) were first sterilized. Each disk was incubated in 2 mL of serum-free α-MEM (corresponding to a surface area-to-volume ratio of ∼0.8 cm^2^/mL) for 24 h at 37 °C under agitation. The resulting extracts were then supplemented with 10% FBS to obtain the final conditioned media (Cp-Ti, CaP, CoCaP). A negative control medium (Ctrl) was prepared identically without any material. For the osteogenic medium (O.M), the control medium was supplemented with osteogenic inducers as described below. For the scanning electron microscopy test, the cells were plated directly on the materials at a density of 5.0 × 10^4^ cells per well and cultivated for 3 and 7 days. No human participants or live animals were directly involved in this study. MC3T3-E1 preosteoblasts cell line used was obtained from certified commercial repositories and handled according to institutional biosafety and ethical guidelines.

### Osteogenic Differentiation

To induce osteogenic differentiation, MC3T3-E1 preosteoblasts were plated at a density of 5 × 10^4^ cells/mL and cultured until reaching ∼50% confluency. Osteogenic differentiation was initiated by supplementing the growth medium with 10 mM β-glycerophosphate, 0.03 μg/mL dexamethasone, and 50 μg/mL ascorbic acid (Sigma-Aldrich). Cells were maintained in this differentiation medium, supplemented with 10% fetal bovine serum (FBS), throughout the experimental period. This condition served as the positive control for osteogenic commitment.

### Cytotoxicity

For cytotoxicity testing, preosteoblasts were plated in 96-well plates and incubated with biomaterial-enriched media for 24 h at a standard cell culture temperature of 37 °C, following the ISO 10993–5:2009 standard for in vitro cytotoxicity tests, as we have widely explored earlier.^7,8^ Post incubation, the medium was replaced with an MTT solution (concentration: 1 mg/mL), and cells were further incubated for 3 h. Following this, the MTT solution was discarded and replaced with DMSO to solubilize the resultant formazan crystals. The optical density of the solution, indicative of cell viability, was quantified at 570 nm using a SYNERGY-HTX multimode microplate reader.

### Cell Adhesion

The cell adhesion assay was conducted using the crystal violet staining technique.^9^ Cells, after routine cultivation and trypsinization, were dispensed into 96-well plates at a density of 1 × 10^5^ cells/mL This assessment was performed to gauge the impact of biomaterial-conditioned media on cell adhesion properties. The cell groups included a control set maintained in standard culture medium and sets exposed to various biomaterials. After a 24-h incubation period, the cells were stained using crystal violet. The degree of cell adhesion was quantitatively estimated by measuring the absorbance of the stained cells at 540 nm, utilizing the SYNERGY-HTX multimode microplate reader.

### Scratch Assay

Cell migration was evaluated using an in vitro scratch assay, as previously described.^10^ This assay provides a simplified model to study cell migration and wound closure. Briefly, preosteoblasts were seeded in 24-well plates at a density of 5 × 10^4^ cells/cm^2^ and cultured to confluence. A linear scratch wound was then introduced into the cell monolayer using a sterile pipet tip. Cell migration and wound closure were monitored at 0 to 24 h postscratch.^10^ During the experiment, the images of the wound area were acquired using an inverted microscope (Zeiss) equipped with a digital camera.

### Quantitative Real-Time PCR (RT-qPCR)

To elucidate the molecular mechanisms involved cellular responses to calcium phosphate substrates, preosteoblasts were exposed to material-conditioned medium for 24 h. Total RNA was extracted using TRIzol reagent (Life Technologies) followed by DNase I treatment (Invitrogen) to eliminate genomic DNA contamination RNA purity was confirmed by measuring the A260/A280 ratio, with all samples yielding ratios between 1.9 and 2.1 (Ctrl: 2.058, O.M: 1.957, cp-Ti: 1.934, CaP: 2.049, CoCaP: 1.964). First-strand cDNA synthesis was performed using the High-Capacity cDNA Reverse Transcription Kit (Applied Biosystems) following the manufacturer’s protocol. Quantitative PCR was carried out using PowerUp SYBR Green Master Mix (Applied Biosystems) in 10 μL reactions containing 5 μL of master mix, 0.4 μM primers, 50 ng cDNA, and nuclease-free water. Gene expression was normalized to the endogenous control β-actin using the 2−ΔΔCt method. Primer efficiencies were validated and found to be between 95% and 105%. Primer sequences and qPCR reaction conditions are provided in Table 1.

**Table 1:** Primer’s Sequences and Details of the Cycle Conditions

gene (ID)	primer	5′-3′ sequence	reaction condition
Runx2 (12393)	Forward	GGACGAGGCAAGAGTTTCA	95 °C—15 s; 60 °C—30 s; 72 °C—60 s
	Reverse	TGGTGCAGAGTTCAGGGAG	
Osterix (170574)	Forward	CCCTTCCCTCACTCATTTCC	95 °C—15 s; 60 °C—30 s; 72 °C—60 s
	Reverse	CAACCGCCTTGGGCTTAT	
α1Integrin (109700)	Forward	TATCCTCCTGAGCGCCTTT	95 °C—15 s; 60 °C—30 s; 72 °C—60 s
	Reverse	TGGCCTTTTGAAGAATCCAA	
β1Integrin (16412)	Forward	CTGATTGGCTGGAGGAATGT	95 °C—15 s; 60 °C—30 s; 72 °C—60 s
	Reverse	TGAGCAATTGAAGGATAATCATAG	
Fak (14083)	Forward	TCCACCAAAGAAACCACCTC	95 °C—15 s; 60 °C—30 s; 72 °C—60 s
	Reverse	ACGGCTTGACACCCTCATT	
Src (20779)	Forward	TCGTGAGGGAGAGTGAGAC	95 °C—15 s; 60 °C—30 s; 72 °C—60 s
	Reverse	GCGGGAGGTGATGTAGAAAC	
Cofilin (12631)	Forward	CAGACAAGGACTGCCGCTAT	95 °C—15 s; 60 °C—30 s; 72 °C—60 s
	Reverse	TTGCTCTTGAGGGGTGCATT	
HIF1α (15251)	Forward	CATAAAGTCTGCAACATGGAAGGT	95 °C—15 s; 60 °C—30 s; 72 °C—60 s
	Reverse	ATTTGATGGGTGAGGAATGGGTT	
VEGF (22339)	Forward	TGCAGATTATGCGGATCAAACC	95 °C—15 s; 60 °C—30 s; 72 °C—60 s
	Reverse	TGCATTCACATTTGTTGTGCTGTAG	
Bmp2 (12156)	Forward	GGT CAC AGC TAA GGC CAT TGC	95 °C—15 s; 60 °C—30 s; 72 °C—60 s
	Reverse	GCT TCC GCT GTT TGT GTT TG	
eNOS (18127)	Forward	AAG CCG CAT ACG CAC CCA GAG	95 °C—15 s; 60 °C—30 s; 72 °C—60 s
	Reverse	TGG GGT ACC GCT GCT GGG AGG	
MMP2 (17390)	Forward	AAC TTT GAG AAG GAT GGC AAG T	95 °C—15 s; 60 °C—30 s; 72 °C—60 s
	Reverse	TGC CAC CCA TGG TAA ACA A	
MMP9 (17395)	Forward	TGT GCC CTG GAA CTC ACA CGA C	95 °C—15 s; 60 °C—30 s; 72 °C—60 s
	Reverse	ACG TCG TCC ACC TGG TTC ACC T	
β-Actin (11461)	Forward	TCT TGG GTA TGG AAT CCT GTG	95 °C—15 s; 60 °C—30 s; 72 °C—60 s
	Reverse	AGG TCT TTA CGG ATG TCA ACG	
Col1a1 (12842)	Forward	CAC TGG TGA TGC TGG TCC TG	95 °C—15 s; 60 °C—30 s; 72 °C—60 s
	Reverse	CGA GGT CAC GGT CAC GAA C	
Col3a1 (12825)	Forward	GAC CTG AAA TTC TGC CAT CC	95 °C—15 s; 60 °C—30 s; 72 °C—60 s
	Reverse	GCA TGT TTC CCC AGT TTC C	
CDK2 (12566)	Forward	TAC CCA GTA CTG CCA TCC GA	95 °C—15 s; 60 °C—30 s; 72 °C—60 s
	Reverse	CGG GTC ACC ATT TCA GCA AA	
CDK4 (12567)	Forward	TCG ATA TGA ACC CGT GGC TG	95 °C—15 s; 60 °C—30 s; 72 °C—60 s
	Reverse	TTC TCA CTC TGC GTC GCT TT	
CDK6 (12571)	Forward	CGC CGA TCA GCA GTA TGA GT	95 °C—15 s; 60 °C—30 s; 72 °C—60 s
	Reverse	GCC GGG CTC TGG AAC TTT AT	

### MMPs Activity for Zymography

Matrix metalloproteinase (MMP) activity in conditioned media was evaluated by gelatin zymography. Samples were centrifuged at 14,000*g* for 15 min to remove cellular debris, and total protein concentration was determined using the Lowry assay. Equal protein amounts were resolved by electrophoresis on 12% SDS-polyacrylamide gels copolymerized with 4% gelatin. Postelectrophoresis, gels were incubated in 2% Triton X-100 to renature proteins, followed by overnight incubation in Tris-CaCl_2_ buffer (pH 7.4) at 37 °C to facilitate gelatinolysis. Gels were stained with 0.05% Coomassie Blue R-250 and destained in 30% methanol/10% acetic acid. Clear proteolytic bands corresponding to MMP-2 (62 kDa) and MMP-9 (84 kDa) were densitometric-quantified using ImageJ (NIH).

## Results

The sample denominations were as follows: c.p.-Ti + CaP for the titanium samples coated with monetite and c.p.-Ti + CoCaP for the titanium samples where monetite was augmented with cobalt. When examined structurally, both the coated and the original titanium materials displayed XRD spectra consistent with reference patterns from the standard database: entry #71–1760 for Monetite (CaHPO_4_) associated with the coated samples, and entry #44–1294 corresponding to the unaltered titanium. These findings are depicted in Figure 1, with the corresponding crystallographic details (crystal structure, spatial group, and cell parameters) provided in Table 2. The phase composition and cell parameters obtained from the Rietveld refinement are presented in Table 3, which indicates that the α-Ti crystalline structure was primarily observed for the cp-Ti, whereas monetite was fully observed in the coated samples. Co was incorporated as substitutional element, changing the cell parameters instead of the phase composition. The presence of Co tended to reduce the cell parameters of monetite, resulting in a smaller cell volume than in the undoped sample. Lastly, the corresponding crystallite size and microstrain for the monetite and Co-doped monetite are shown in Table 4. The crystallite size estimated by the Scherrer equation (Ds) did not show clear differences with Co incorporation, remaining in the order of dozens of nanometers. However, the value estimated using the Williamson–Hall equation (Dwh) decreased, with a subsequent increase in compressive microstrain, indicating that the incorporation of minor amounts of Co into monetite resulted in discernible crystalline effects on the cell lattice. These findings are depicted in Figure 1.

**Figure 1:**
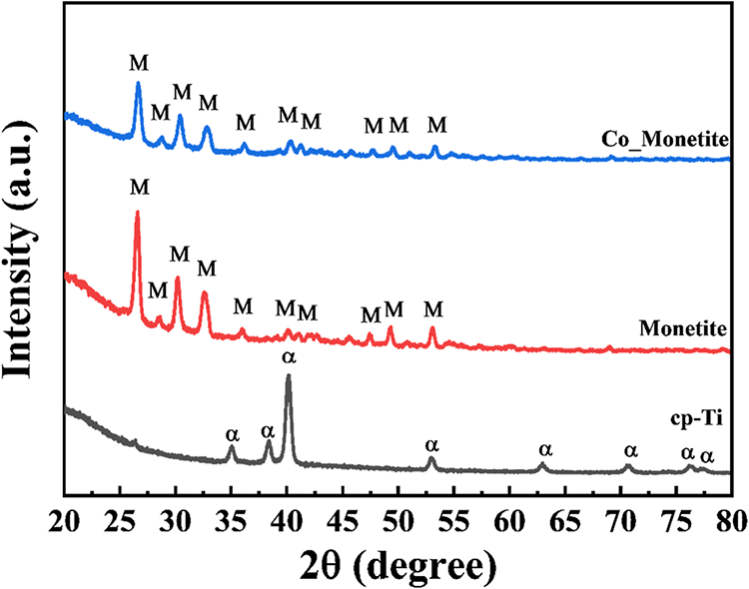
Depicts the X-ray diffraction patterns of the samples, in order c.p.-Ti heated to 200 °C, c.p.-Ti with a CaP coating, and c.p.-Ti with a CoCaP coating. Detailed crystallographic information, including lattice and unit cell parameters, as well as data pertaining to the crystallite size and microstrain within the Monetite phase, are collated in Tables 2–4.

**Table 2:** Structural Details of the Crystallographic Datasheets

phase	monetite	α-Ti
nomenclature	M	α
chemical composition	CaHPO_4_	Ti
CIF number	ICSD #10503	ICSD #43416
crystal structure	triclinic	hexagonal
spatial group	*P*1̅	*P*_63_/mmc
cell parameters	α = 96.18°	α = 90°
	β = 103.82°	β = 90°
	γ = 88.34°	γ = 120°
	*a* = 6.910 Å	*a* = 2.951 Å
	*b* = 6.619 Å	*b* = 2.951 Å
	*c* = 6.998 Å	*c* = 4.684 Å

**Table 3:** Cell Parameters

sample	phase* (%)	α (°)	β (°)	γ (°)	*a* (Å)	*b* (Å)	*c* (Å)	*V* (Å^3^)
cp-Ti	α—100	90	90	120	2.953 (1)	2.953 (1)	4.687 (1)	35.39 (1)
Monetite	M– > 99	96.2 (1)	104.0 (1)	88.4 (1)	6.906 (1)	6.626 (1)	6.999 (1)	308.5 (1)
	α– < 1							
Co doped Monetite	M– > 99	96.2 (1)	104.0 (1)	88.4 (1)	6.868 (1)	6.599 (4)	6.959 (4)	304.2 (5)
	α– < 1							

**Table 4:** Crystallite and Microstrain of Monetite Phase

sample	phase	Ds (nm)	Dwh (nm)	ε (%)
cp-Ti	α	12.8 (2)	27.7 (2)	– 0.04 (4)
monetite	M	41.1 (9)	58.3 (4)	–0.01 (1)
Co doped monetite	M	41.4 (6)	54.2 (4)	–0.75 (2)

Morphological characterization of the surfaces was performed through scanning electron microscopy (SEM) and surface profilometry, as illustrated in Figure 2. Concurrently, Figure 3 depicts the interaction between the cells and the surfaces through SEM micrographs, highlighting the details of cellular morphology. The morphological analyses of the surfaces were carried out using SEM and profilometry (Figure 2) and Figure 3 brings micrographs of the cells interacting with the surfaces, highlighting the cell morphology changes. The EDS analyses showed the homogeneous distribution of cobalt in the sample (Figure 2). Quantitative profilometry confirmed the significant alteration of surface topography induced by the coatings (Figure 2d–f). The average roughness (*R*_a_) increased dramatically from 0.398 μm for the polished cp-Ti substrate to 7.674 μm for the monetite (CaP) coating. The cobalt-doped monetite (CoCaP) coating exhibited a slightly reduced yet still high Ra value of 5.246 μm. A similar trend was observed for the root-mean-square roughness (*R*_q_), with values of 0.511 μm (cp-Ti), 11.281 μm (CaP), and 6.666 μm (CoCaP). The maximum peak height (*R*_p_) also followed this pattern, measuring 3.599 μm for cp-Ti, 32.928 μm for CaP, and 27.198 μm for CoCaP. These data quantitatively demonstrate that both CaP and CoCaP hydrothermal coatings create a microstructured surface with high roughness, a key parameter known to influence osteoblast response.

**Figure 2:**
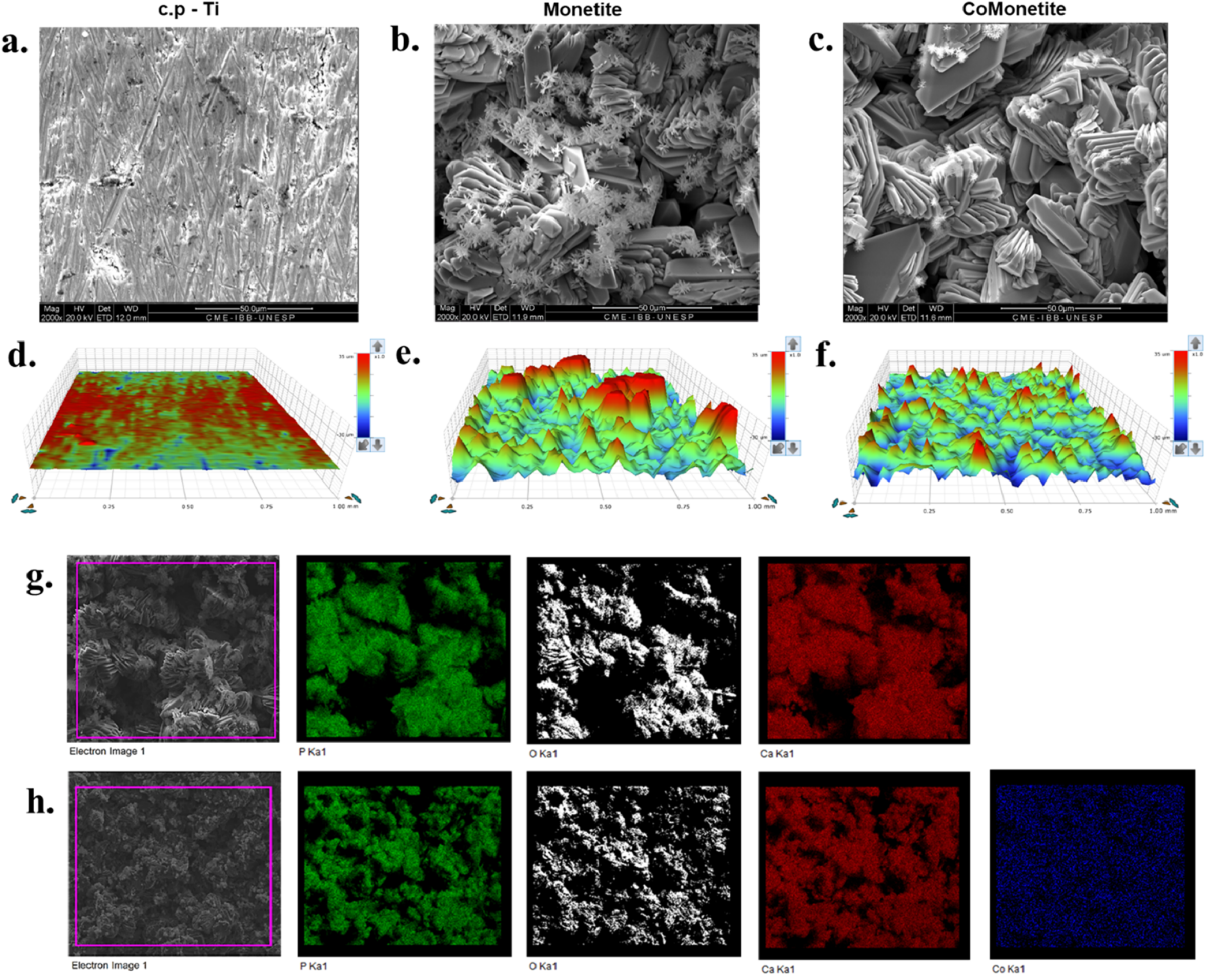
Surface characterization of titanium samples. Scanning Electron Microscopy (SEM) images showing the texture and morphology of (a) uncoated commercially pure titanium (cp-Ti), (b) cp-Ti with a Monetite coating, and (c) cp-Ti with a Cobalt-doped Monetite (Co-Monetite) coating. Profilometry scans depicting the surface topography of (d) uncoated cp-Ti, (e) Monetite-coated cp-Ti, and (f) Co-Monetite-coated cp-Ti, illustrating the variations in surface roughness and structure postcoating application. Semiquantitative analysis and elemental distribution of cobalt-doped by EDS. (g) Monetite; (h) Co-doped Monetite.

**Figure 3:**
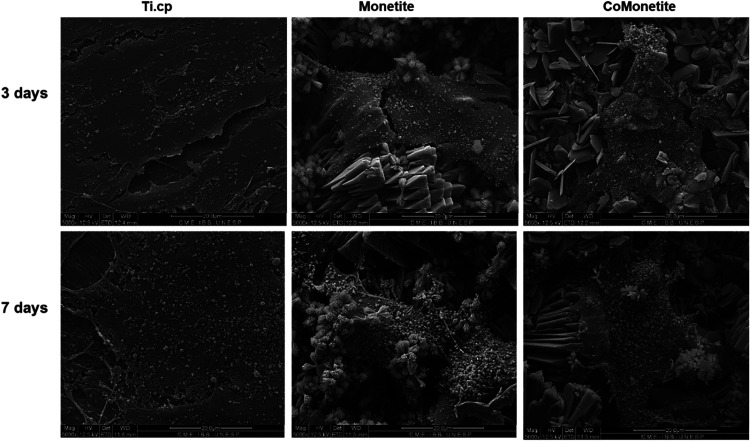
Osteoblast morphology on different titanium surfaces over time. This series of SEM micrographs captures the growth pattern of cells after 3 and 7 days of direct contact with (a) untreated commercially pure titanium (cp-Ti), (b) cp-Ti coated with Monetite, and (c) cp-Ti coated with Cobalt-doped Monetite (Co-Monetite). The images illustrate the influence of surface microtopography on cell morphology, with differences becoming more pronounced over the course of the observation period.

Thereafter, the Figure 3 presents the SEM micrographs depicting the cellular architecture as the cells proliferate in direct adherence with the various material surfaces. Notably, changes in cell morphology were observed, which can be correlated to the varying degrees of surface roughness provided by each type of evaluated coating.

The contact angle analysis revealed an enhancement in hydrophilicity across the various coatings, as delineated in Figure 4. The recorded contact angles of water droplets on the surfaces exhibit discernible differences, reflecting the impact of the coatings. Dynamic mode measurement was employed to capture the interaction between the droplet and the surface in real-time, enabling the determination of the initial contact angle precisely upon droplet placement. The coated materials showed high hydrophilicity, so the average possible angle of detection was 11.00° for the CaP-coated material and 10.10° for CoCaP, while for the uncoated material it was 90.10°.

**Figure 4:**
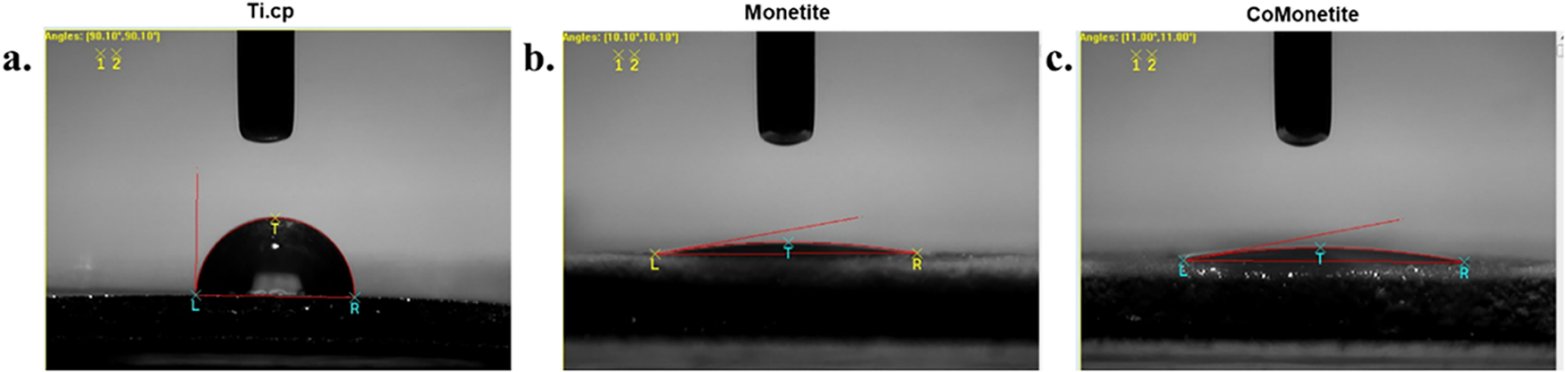
Evaluation of surface hydrophilicity through contact angle measurements. Panel (a) illustrates the contact angle on uncoated commercially pure titanium (cp-Ti), while panels (b, c) depict the contact angles on Monetite-coated cp-Ti and Cobalt-doped Monetite (Co-Monetite)-coated cp-Ti, respectively. The snapshots in panels (b, c) were captured approximately 0.099 s after the water droplet made contact with the coatings, highlighting the immediate wetting response due to the surface modifications.

The metabolic activity of MC3T3-E1 cells was significantly enhanced by CaP and CoCaP conditioned media compared to the control (Figure 5a).

**Figure 5:**
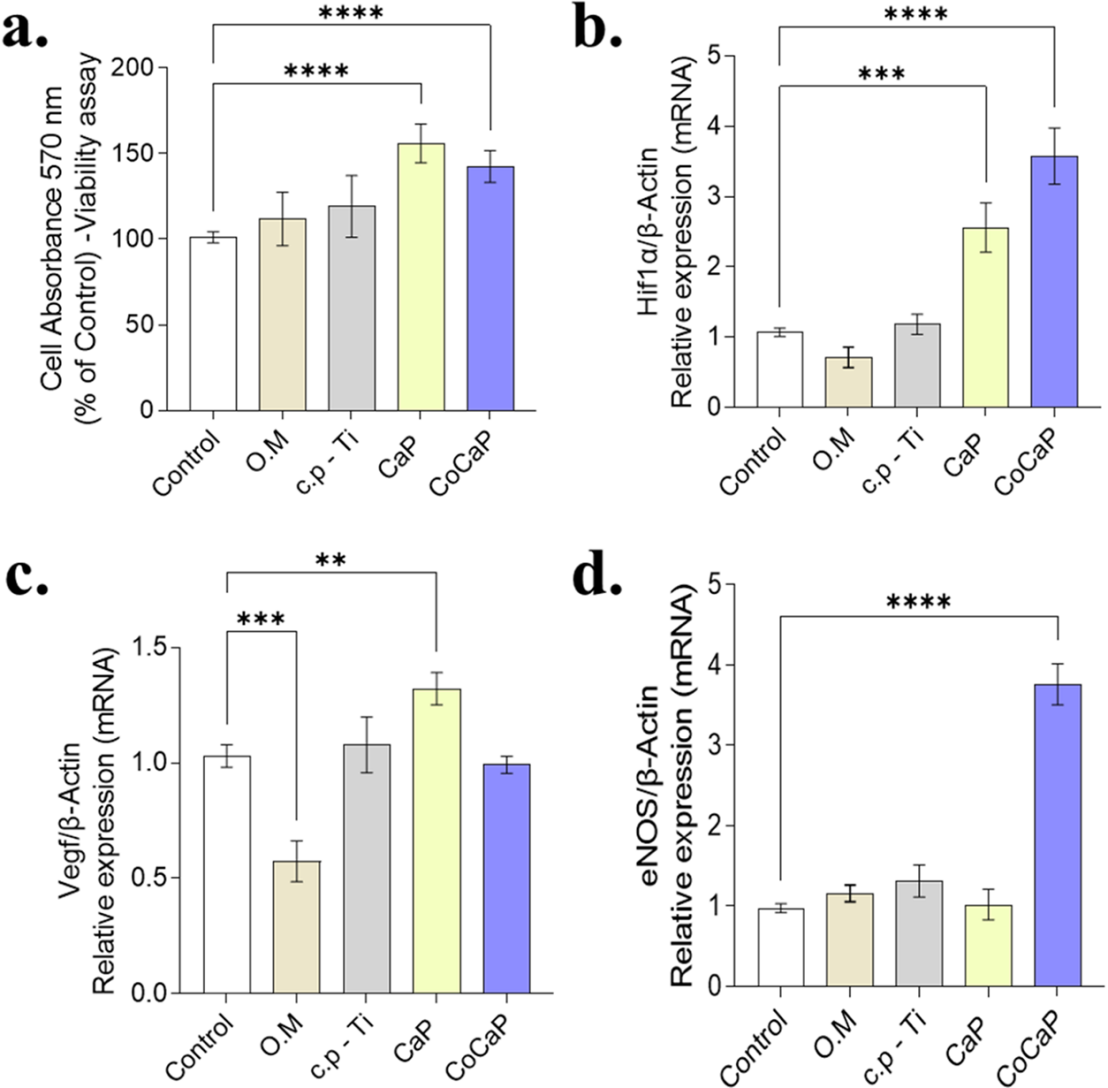
Effect of conditioned media on cell viability and expression of hypoxia, angiogenesis, and vasoregulation-related genes. (a) Cell viability was significantly increased in CaP and CoCaP groups compared to control (*p* < 0.0001). (b) Hif1α expression showed significant upregulation in CaP (*p* = 0.0002) and CoCaP (*p* < 0.0001) groups versus control. (c) VEGF expression was significantly higher in CaP (*p* = 0.0075) and O.M (*p* = 0.0003) groups compared to control. (d) eNOS expression was modulated in CoCaP group versus all other treatments (*p* < 0.0001), while showing no significant changes in other comparisons (all *p* > 0.05). Data are presented as mean ± SD of *n* = 3 independent biological experiments. Statistical significance was determined by one-way ANOVA followed by the appropriate posthoc test (Tukey’s or Dunnett’s). Exact p-values and detailed comparisons are indicated in the figure (*p < 0.05, **p < 0.01, ***p < 0.001, ****p < 0.0001).

Gene expression analysis revealed significant modulation of pathways related to hypoxia and angiogenesis (Figure 5). Hif1α expression was strongly upregulated in cells treated with CaP and CoCaP conditioned media compared to control. VEGF expression was also significantly increased in the CaP and O.M groups. Notably, eNOS expression was markedly induced only in the CoCaP group, showing a highly significant difference from all other treatments.

Cell cycle progression and migration capacity were evaluated (Figure 6). The scratch assay confirmed that none of the conditioned media impaired the wound closure ability of MC3T3-E1 cells. Analysis of cell cycle regulators revealed a complex, treatment-specific modulation. Cdk2 expression was suppressed in most groups but increased in the CoCaP condition. Cdk4 was upregulated by O.M and CoCaP treatments, while Cdk6 expression was generally decreased across all material-conditioned groups except O.M.

**Figure 6:**
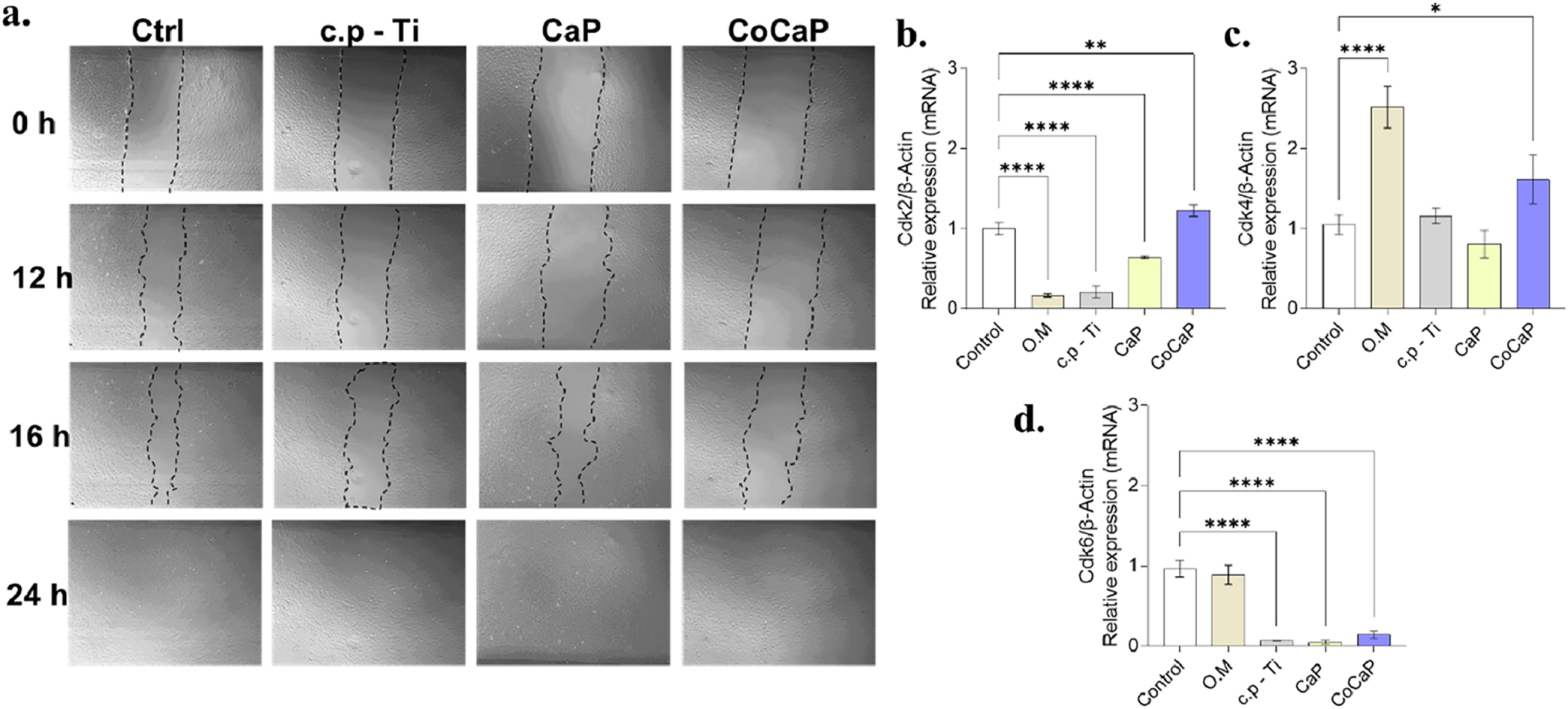
Effect of conditioned media on cell proliferation and cell cycle gene expression in MC3T3-E1 cells. (a) Scratch assay demonstrated enhanced wound closure at 24 h compared to baseline (0 h). (b) Cdk2 expression was significantly upregulated in the CaP group versus control (*p* < 0.0001). (c) Cdk4 expression showed a marked increase in the CoCaP group (*p* = 0.0001) compared to control. (d) Cdk6 expression was significantly higher in both CaP (*p* = 0.0032) and CoCaP (*p* < 0.0001) groups versus control. Data are presented as mean ± SD of *n* = 3 independent biological experiments. Statistical significance was determined by one-way ANOVA followed by the appropriate posthoc test (Tukey’s or Dunnett’s). Exact *p*-values and detailed comparisons are indicated in the figure (*p < 0.05, **p < 0.01, ***p < 0.001, ****p < 0.0001).

The molecular machinery governing cell adhesion and cytoskeletal dynamics was significantly affected (Figure 7). Functionally, only the CoCaP-conditioned medium led to a measurable increase in cell adhesion. At the molecular level, this was consistent with the strong upregulation of key adhesion-related genes (Integrin α1, Integrin β1, and FAK) specifically in the CoCaP group. Furthermore, CoCaP treatment uniquely induced a dramatic increase in Cofilin expression, suggesting active cytoskeletal remodeling. Src expression showed a more variable pattern across different treatments.

**Figure 7:**
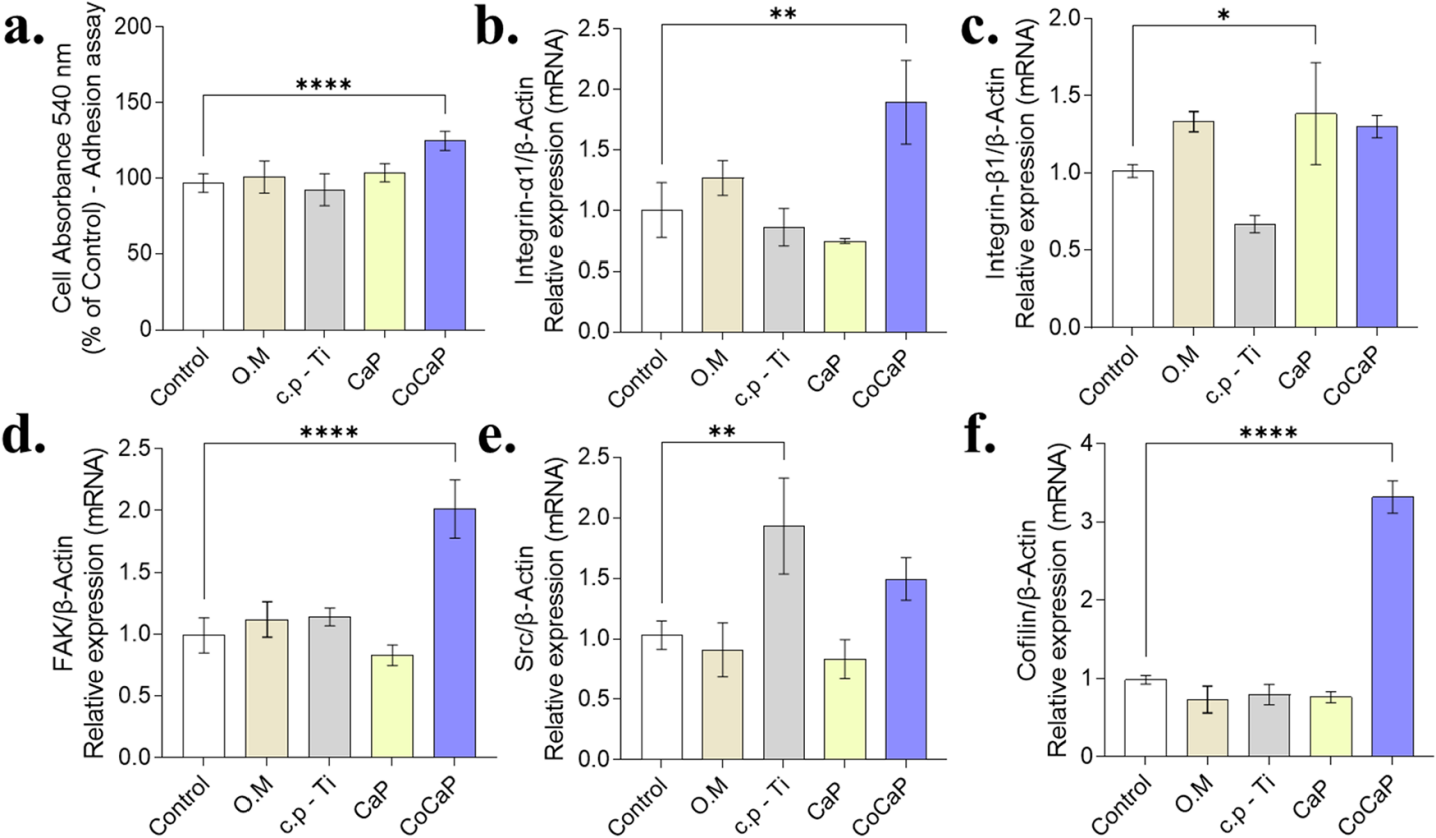
Treatment effects on cell adhesion and extracellular matrix/cytoskeleton-related gene expression. (a) The CoCaP group showed significantly increased cell adhesion compared to control (*p* < 0.0001). (b) Integrin α1 expression was significantly higher in CoCaP group versus control (*p* = 0.0027). (c) Integrin β1 showed significant differences between c.p-Ti and CaP groups (*p* = 0.0017). (d) FAK expression was increased in CoCaP group (*p* < 0.0001). (e) Src expression was higher in c.p-Ti group versus control (*p* = 0.0060). (f) Cofilin showed significantly elevated expression in CoCaP group compared to others (*p* < 0.0001). Data are presented as mean ± SD of *n* = 3 independent biological experiments. Statistical significance was determined by one-way ANOVA followed by the appropriate posthoc test (Tukey’s or Dunnett’s). Exact p-values and detailed comparisons are indicated in the figure (*p < 0.05, **p < 0.01, ***p < 0.001, ****p < 0.0001, ns: not significant).

The expression profile of major osteogenic markers and extracellular matrix (ECM) components highlighted clear differences in osteogenic induction (Figure 8). Runx2, a master transcription factor for osteoblast differentiation, was strongly upregulated only by the CoCaP treatment. In contrast, Osterix expression showed minimal variation. BMP2 was significantly induced by all material-conditioned media, with the strongest effect observed for c.p-Ti and CaP. Similarly, the expression of the collagen genes Col1a1 and Col3a1, essential for bone matrix formation, was markedly enhanced by most treatments, with CoCaP showing a particularly strong effect on Col3a1.

**Figure 8:**
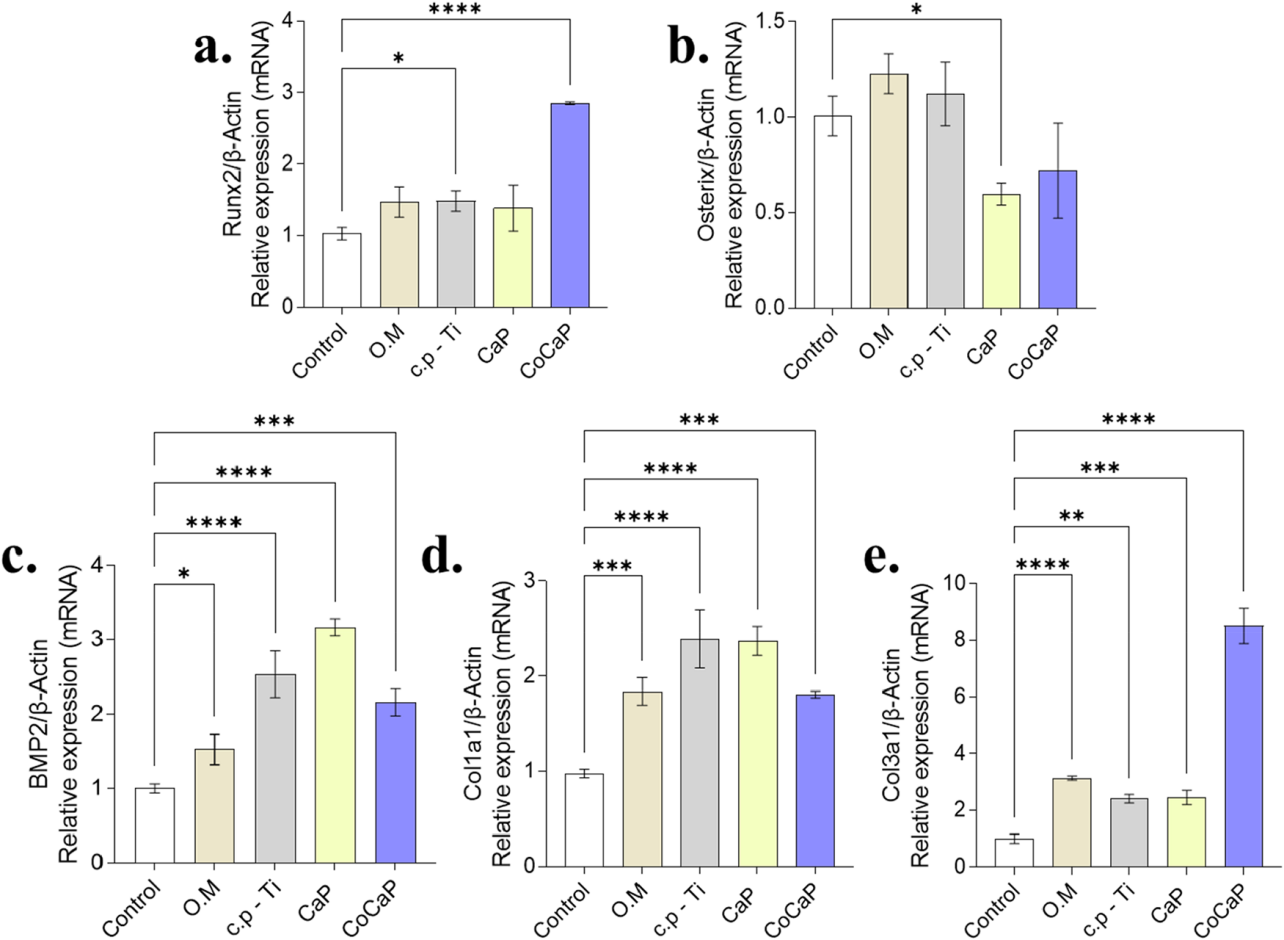
Expression of osteogenic markers and extracellular matrix components. (a) Runx2 expression was significantly increased in CoCaP group (*p* < 0.0001). (b) Osterix showed significant difference only between CaP and O.M groups (*p* = 0.0086). (c) BMP2 expression was modulated by different treatments. (d) Col1a1 expression was significantly increased in c.p-Ti and CaP groups (*p* < 0.0001). (e) Col3a1 showed elevated expression in CoCaP group (*p* < 0.0001). Data are presented as mean ± SD of *n* = 3 independent biological experiments. Statistical significance was determined by one-way ANOVA followed by the appropriate posthoc test (Tukey’s or Dunnett’s). Exact p-values and detailed comparisons are indicated in the figure (*p < 0.05, **p < 0.01, ***p < 0.001, ****p < 0.0001).

MMP2 and MMP9 expression and activity were also modulated. While MMP2 gene expression showed limited changes, its enzymatic activity was significantly higher in all treated groups (*p* < 0.0001). MMP9, however, exhibited both increased expression (*p* < 0.0001 in CoCaP) and activity (*p* < 0.0001 in c.p - Ti and CaP), indicating a strong role in ECM degradation (Figure 9).

**Figure 9:**
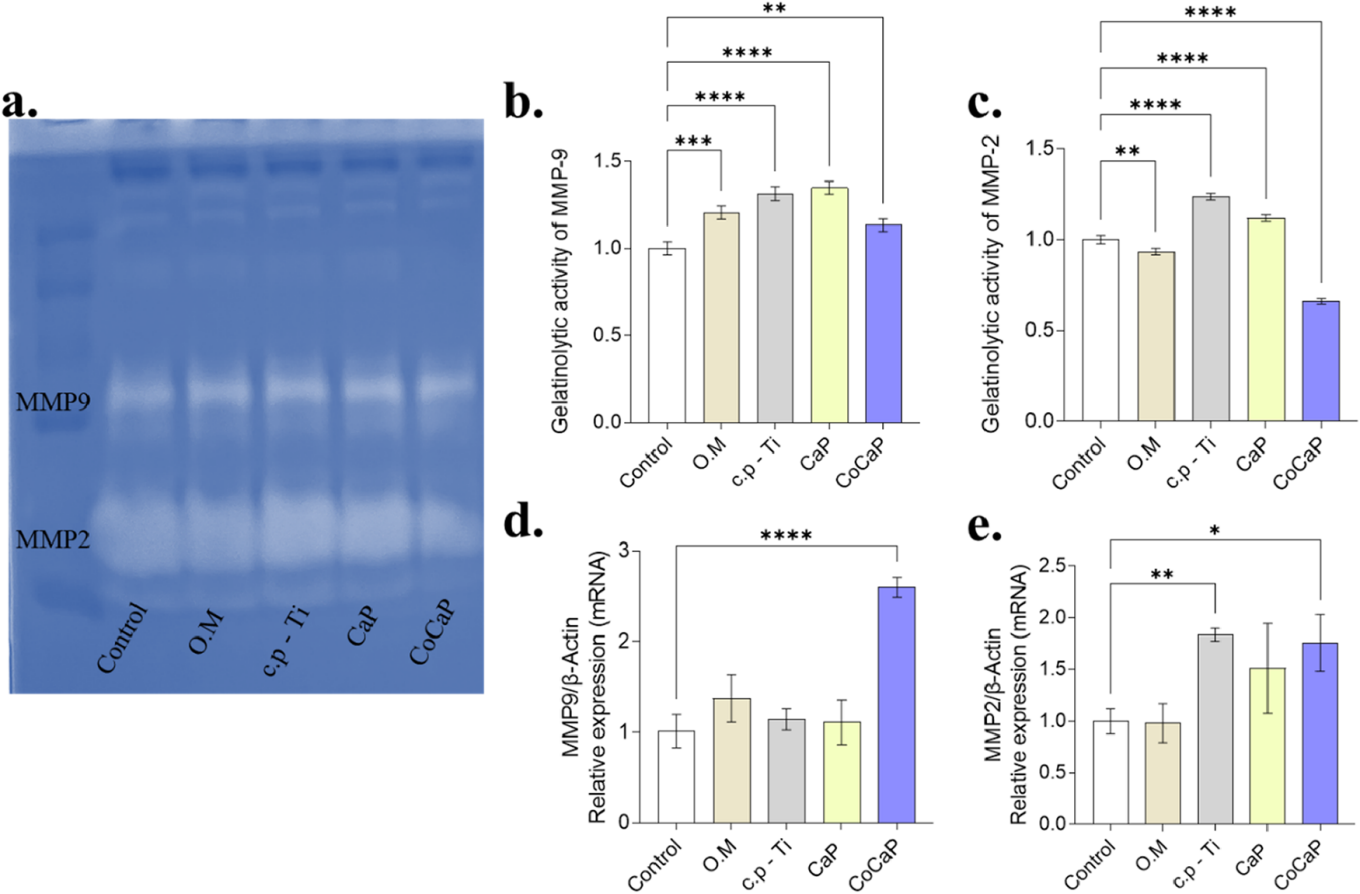
MMP activity and expression. (a) Zymography gel showing MMP2 and MMP9 activity. (b) MMP9 activity was significantly higher in c.p-Ti and CaP groups (*p* < 0.0001). (c) MMP2 activity was increased in all treated groups (*p* < 0.0001). (d) MMP9 expression was significantly higher in CoCaP group (*p* < 0.0001). (e) MMP2 expression was increased in CoCaP group (*p* = 0.0274). Data are presented as mean ± SD of *n* = 3 independent biological experiments. Statistical significance was determined by one-way ANOVA followed by the appropriate posthoc test (Tukey’s or Dunnett’s). Exact *p*-values and detailed comparisons are indicated in the figure (*p < 0.05, **p < 0.01, ***p < 0.001, ****p < 0.0001).

## Discussion

This study highlights the physicochemical attributes of monetite and cobalt-doped monetite coatings on titanium surfaces and their subsequent impact on osteoblast activity. The XRD analysis reveals the formation of the monetite phase, consistent with the established literature, demonstrating that Co incorporation does not disrupt the structural integrity of monetite, a factor crucial for biological efficacy. The formation of this phase is pivotal, as previous studies have shown that the crystalline structure of surface coatings can modulate cellular responses. Furthermore, the structural analysis demonstrated that Co addition affected the cell parameters. The decrease in cell parameters results from the smaller ionic radius of Co^2+^ (∼0.745 Å) replacing Ca^2+^ (∼1.000 Å) in the monetite crystal structure, thereby promoting a contraction of the cell volume. In addition, because the Williamson-Hall approach accounts for microstrain, it is more effective at detecting structural defects in monetite containing minor amounts of Co than the Scherrer relation. The decrease in crystallite size and the variation in compressive microstrain indicate that Ca replacement by Co modifies local chemical bonding within the crystal structure, resulting in a compacted and more strongly bonded monetite. Overall, previous studies have shown that it produces discernible effects on HPO_4_^2–^ groups, altering the thermal stability and phase transition of monetite,^2^ as well as its stability in aqueous media and its transformation into apatite upon contact with SBF.^11^

Our findings align with recent studies demonstrating that surface topography and chemistry dictate osteoblast fate.^12,13^ The profilometry data provide quantitative confirmation of the microstructured topography observed by SEM. The Ra values increased from 0.398 μm (smooth cp-Ti) to 7.674 μm for CaP and 5.246 μm for CoCaP coatings, placing them well within the microscale roughness range (1–10 μm) known to be optimal for osteoblast adhesion and osteogenic differentiation.^14,15^ The Rq and Rp values followed the same trend. While both coatings significantly increased roughness compared to polished titanium, the slightly lower values for CoCaP suggest that cobalt doping might subtly influence the nucleation and growth kinetics of the monetite crystals during hydrothermal synthesis, leading to a marginally different surface architecture. This created topography, featuring peaks and valleys, directly mimics the native bone morphology and provides an enlarged surface area for protein adsorption and cell attachment. The hydrophilicity (contact angles ∼ 10°) of the CaP and CoCaP coatings is intrinsically linked to their high surface roughness. The microstructured topography, with Ra values exceeding 5 μm, promotes capillary action and complete wetting of the surface.^16^ This synergistic combination of high roughness and superhydrophilicity is crucial, as it maximizes the initial interactions with the biological fluid, enhancing the adsorption of adhesive proteins like fibronectin and vitronectin from the serum. This, in turn, creates a favorable interface for the observed enhancement in preosteoblast adhesion, spreading (Figure 3), and the upregulation of integrin and FAK signaling (Figure 7), effectively priming the surface for subsequent osteogenic events.

The SEM observations illustrate a topography that not only emulates natural bone growth but also triggers osteogenic signals, affirming the findings of Zhou et al.^1^ and Zavgorodniy et al.^17^ who reported similar morphological influences on cell function. Regarding the plate-like and whiskers structures we observe parallel natural osseous formations, which have been shown to enhance osteoblast adhesion and proliferation.^18,19^ Our EDS data add to the growing body of evidence that the homogeneity of elemental distribution is critical in biomedical coatings, with cobalt’s role in angiogenesis and bone growth being particularly emphasized.^20^ Additionally, SEM analyses of cellular interactions align with recent investigations,^1,17,21–23^ which highlighted the role of the microenvironment in directing cell fate. The thriving osteoblasts on our coatings are not only evidence of viability but also functional activity, suggesting that the Monetite and Co-doped Monetite surfaces act as more than passive substrates; they serve as active participants in the bone regeneration process.

Profilometry results further elaborate on the surface characteristics, suggesting an optimal roughness that aligns with the study of Zavgorodniy et al.,^24^ who found that specific topographical scales could upregulate osteogenic differentiation markers. Our findings contribute to this discourse by demonstrating that such microscale roughness can be achieved with Monetite coatings, enhancing the material’s bioactivity.

The increase in hydrophilicity observed in our coatings correlates with a growing consensus in the literature regarding the importance of surface wettability in osseointegration.^25,26^ Hydrophilic surfaces have been consistently linked with improved protein adsorption and initial cell interaction, a key for the successful integration of implants.^13,27,28^ This study aligns with ISO10993 standards for biological assays reaffirms the coatings’ biocompatibility and supports their potential for clinical application or at least in preclinical evaluations.

The viability and adhesion assays demonstrate that while both CaP and CoCaP enhance cellular metabolic activity, only CoCaP significantly improves cell adhesion properties, indicating distinct mechanisms of action for these treatments. The differential effects on viability versus adhesion highlight the importance of evaluating multiple cellular parameters when assessing biomaterial treatments.

The gene expression results for Cdk2, Cdk4, and Cdk6 in MC3T3-E1 cells provide important information about the impact of the tested materials on cell proliferation. The fact that the conditioned medium did not impair MC3T3-E1 cell proliferation in the scratch assay is consistent with the modulation of these genes’ expression. The decreased expression of Cdk2 in the O.M, c.p-Ti, and CaP groups suggests a possible reduction in the cell cycle progression rate under these conditions, although overall proliferation was not compromised in the functional assay. The increased Cdk2 expression in the CoCaP group may indicate a stimulus for cell cycle progression. The elevation of Cdk4 in the O.M and CoCaP groups also points to a promotion of S phase entry under these conditions. The generalized decrease in Cdk6, except in O.M, may indicate a differentiated regulatory pathway, where Cdk4 plays a more prominent role or other compensatory mechanisms are at play. In osteoblastic cells like MC3T3-E1, proper cell cycle regulation is fundamental for differentiation and mineralization. The absence of severe proliferative inhibition, as suggested by the functional results, combined with the modulation of Cdk expression, indicates a biocompatible profile of the materials concerning cell kinetics.^29,30^ The observed modulation in Cdks suggests that the materials can influence cell cycle dynamics in specific ways, potentially optimizing proliferation or preparing cells for subsequent differentiation processes, without, however, inhibiting overall proliferative capacity.^9,31^

The observed upregulation of FAK and Integrins in the CoCaP group suggests enhanced cell-ECM interactions, which are crucial for osteoblast adhesion and differentiation.^32,33^ The significant increase in Cofilin further supports cytoskeletal remodeling, potentially facilitating cell migration and matrix reorganization.^34,35^

The significant induction of Col1a1 and Col3a1 in CoCaP-treated cells (both *p* < 0.0001) indicates enhanced matrix production, while the treatment-specific patterns of integrin expression suggest differential engagement with matrix components.^36,37^ These changes likely contribute to the observed enhancement of cell adhesion in CoCaP-treated groups and may facilitate subsequent differentiation events. However, the stark upregulation of MMP2/9 activity suggests a simultaneous increase in matrix degradation, which may reflect a dynamic remodeling process necessary for osteogenesis.^38,39^ The discrepancy between MMP2 gene expression and activity hints at post-translational regulation, whereas MMP9 appears to be transcriptionally controlled.

These preliminary results provide a comprehensive molecular characterization of preosteoblast responses to conditioned media treatments, revealing distinct patterns of osteogenic regulation. The observed upregulation of Runx2 in CoCaP-treated cells (*p* < 0.0001), coupled with minimal changes in Osterix expression, suggests that this treatment preferentially activates early stages of osteoblast commitment rather than terminal differentiation.^40–42^ This finding is particularly significant as Runx2 is known to be a master regulator of osteoblast lineage determination, while Osterix functions downstream in the differentiation cascade.^43^

The differential expression patterns of BMP2 across treatment groups offer important insights into the mechanisms of osteoinduction.^44–46^ The robust upregulation of BMP2 in both c.p-Ti and CaP groups (*p* < 0.0001) indicates these treatments may act through classical BMP signaling pathways to stimulate osteogenesis. In contrast, CoCaP treatment produced a more moderate increase in BMP2 expression (p = 0.0002) while still strongly inducing Runx2, suggesting the involvement of alternative or complementary signaling mechanisms.^46^ This observation is consistent with emerging evidence that certain biomaterials can activate osteogenic programs through BMP-independent pathways.^47–49^

The unique upregulation of eNOS specifically in CoCaP-treated cells (*p* < 0.0001) has important implications for bone regeneration strategies.^50^ As eNOS-derived nitric oxide plays crucial roles in both angiogenesis and osteoblast activity, this finding suggests that CoCaP treatment may create a more favorable microenvironment for bone formation by simultaneously promoting vascularization and osteogenesis.^51–55^ This dual effect could be particularly advantageous in clinical scenarios requiring rapid vascularization of regenerating bone tissue.

The hypoxic response via Hif1α and VEGF may indicate adaptive mechanisms to promote angiogenesis, essential for bone repair.^56–59^ The dual osteogenic-angiogenic response mirrors observations in hypoxia-mimicking biomaterials.^60^ Notably, eNOS induction (Figure 6d) suggests Co-monetite promotes NO-mediated vasodilation, a mechanism critical for implant vascularization.^61^ However, the modest Osterix response (Figure 8b) implies Co-monetite favors early osteoblast commitment over terminal differentiation, a nuance requiring further study.

While the pro-angiogenic effect of cobalt is advantageous, its release kinetics and local concentration are critical for safety. In our system, cobalt is incorporated into the stable monetite lattice, which is expected to modulate its release compared to free ions or soluble salts. Studies suggest that low, sustained release of Co^2+^ (in the micromolar range) can stimulate HIF-1α stabilization and angiogenesis without presenting any cytotoxicity. However, future studies quantifying Co^2+^ release kinetics via ICP-MS are essential to confirm the behavior of Co in biological systems, defining the microenvironment concentration window, and fully map the translational potential of this coating strategy. Summarizing, we successfully developed Co-doped monetite coatings on titanium via a hydrothermal method reflecting on combined osteoconductive properties of monetite with the pro-angiogenic stimulus of cobalt looking for creating a biomimetic microenvironment for bone regeneration during osseointegration.
